# Time perspective and family history of alcohol dependence moderate the effect of depression on alcohol dependence: A study in Chinese psychiatric clinics

**DOI:** 10.3389/fpsyg.2022.903535

**Published:** 2022-10-28

**Authors:** Haiyan Wang, Yichen Zhu, Jie Shi, Xiaoyu Huang, Xiaoying Zhu

**Affiliations:** ^1^Affiliated Mental Health Center & Hangzhou Seventh People's Hospital, Zhejiang University School of Medicine, Hangzhou, China; ^2^Zhejiang University School of Medicine, Hangzhou, China; ^3^School of Life Science, Brain Mind Institute, École Polytechnique Fédérale de Lausanne (EPFL), Lausanne, Switzerland

**Keywords:** alcohol use disorder, alcohol dependence, time perspective, depression, ZTPI

## Abstract

**Background:**

Depression and alcohol dependence (AD) are among the most prevalent psychiatric disorders that commonly co-occur. Therefore, gaining a better grasp of factors related to this comorbidity is particularly interesting for clinicians. Past research has highlighted the significant role that time perspective and family history of alcohol dependence (FH) play in the occurrence of depression and AD. However, much remains unexplored in the understanding of the association between them. This study explored how temporal profile and other sociodemographic characteristics of patients diagnosed with AD impact the severity of depression and AD in them.

**Methods:**

This study was multi-centered, including 381 patients. Cross-sectional information was collected from both inpatient and outpatient psychiatric clinics in China. Data were acquired using validated self-report scales, including Michigan Alcoholism Screening Test, Zung Self-Rating Depression Scale, and Zimbardo Time Perspective Inventory-Chinese version. Multiple linear regression analyzes were conducted to control social demographic variables and construct prediction models to inspect the influence factors of variables. Moderation models were constructed to inspect further interplay between variables using hierarchical regression and PROCESS Macro.

**Results:**

Results showed that of all the patients in Chinese psychiatry clinics diagnosed with AD according to the International Classification of Diseases-10, 59.9% met the criteria of depression according to the questionnaire, and time perspective was correlated with the severity of depression. Furthermore, using regression analysis, we found that time perspective and depression could predict AD severity. The moderating role of a past negative time perspective and FH was confirmed between depression and AD. We found that, in our study, only in patients with FH and relatively moderate to high scores of past negative time perspective could the severity of depression predict the severity of AD. Therefore, during the treatment and care of patients with AD, their depression level, time perspective score, and FH should be considered.

## Introduction

The excessive and harmful use of alcohol has been associated with more than 200 diseases, unintentional injuries, and sometimes even death. Studies have found that repeated consumption of alcohol in large quantities over an extended period is detrimental to both physical and mental health. Globally, alcohol is the seventh leading risk factor for poor health, accounting for 4.2% of total disability-adjusted life years (DALY) and 5.2% of deaths in 2016 (World Health Organization, 2019). According to the Global Burden of Disease data in China, alcohol use problems accounted for 18.23 and 2.89% of the DALY attributed to mental and behavioral disorders in men and women, respectively. Overall, alcohol-related diseases are characterized by high prevalence, great harm, and heavy burden of disease (Carvalho et al., 2019). For the diagnostic systems of alcohol-related disorders, the International Classification of Diseases-10 (ICD-10) (World Health Organization, 2004), as well as the Diagnostic and Statistical Manual for Mental Disorders-III (DSM-III) (Cooper and Michels, 1988) and DSM-IV (Guze, 1995) (1980–2013) have distinguished between *alcohol abuse* and *alcohol dependence* (AD) into two distinct disorders, with “abuse” indicating mild and early phase of the illness and “dependence” indicating a more severe manifestation. DSM-V (American Psychiatric Association, 2013) integrates them into a single disorder named *alcohol use disorder* (AUD), with mild, moderate, and severe classifications.

*Depressive disorders* are complex and heterogeneous syndromes characterized by disrupted mood and a series of cognitive and physical symptoms. The association between AD and depressive disorder has been addressed in numerous studies, which suggest a close link. Depressive disorders are the most common psychiatric disorders among people with AD and alcohol abuse (Grant et al., 2004), with the prevalence of depressive disorders greater among those with AD compared to those diagnosed with alcohol abuse. The comorbidity of AD and depression is related to greater severity and worse prognosis than the severity and prognosis for either disorder alone (McHugh and Weiss, 2019). AD has also been associated with the persistence of depressive disorders, whereas alcohol abuse has not (Boschloo et al., 2012).

However, it is still unclear how the two disorders interact. Much of the research unravels the development of co-occurrence. Comorbidity can be explained in at least two ways. First, it could be argued that causal links exist between depression and AD so either depression increases the risk for AD or vice versa. Nevertheless, studies have yielded mixed results. There is evidence of the reciprocal relationship between depression and AD. Studies indicate that depressive symptoms can be caused by excessive alcohol use (Hasin et al., 1996), whereas harmful alcohol use also prolongs the course of depression (Mueller et al., 1994). As for the timing of the first episode of AUD and depression, some studies have found that depressive episodes typically precede the onset of AUD, while others suggest that AUD precedes depressive disorders. Still, others report that the order of onset varies by gender, with women likelier to have an earlier onset of depression than men. Furthermore, persistent depression during abstinence from alcohol is a risk factor for relapse to heavy drinking (Greenfield et al., 1998; Hasin and Grant, 2002).

The second possible explanation is that shared genetic and environmental factors increase susceptibility to both disorders. Common genetic factors that predispose individuals to the concurrence of AD and depression have been sought in family, twin, and general population studies (Kuo et al., 2006), one of which also showed a sex-specific effect (Prescott et al., 2000). Genome-wide association studies (GWASs) have reported genome-wide significant findings for comorbid AD and depression. For example, in a sample of 4,653 African American participants, a genome-wide association at *SEMA3A* gene with comorbid AD and major depression was detected (Zhou et al., 2017). At a neurophysiological level, some fMRI studies indicate that depression and AD are associated with significant disruptions within the reward circuit, which generally serves to guide our attention toward consuming natural rewards (Becker et al., 2017). Among all factors contributing to AUD, genetic factors are the most important, accounting for 60% of the variance, with environmental factors accounting for the remaining 40% (Sarkhel, 2009). A family history of alcohol dependence generally predicts the presence of AD among probands. Moreover, studies have supported the notion that a positive family history of AD may be associated with a higher risk for AD and contribute to a higher probability of psychiatric disorders other than AD, such as depressive disorders (Sjoerds et al., 2013). A family history of AUD in patients with AD would lead to severe physical problems and high levels of antisocial behavior (Milne et al., 2009). One GWAS study based on molecular genetic information supported the causal role of genetic liability of depression on AD instead of AD on depression (Polimanti et al., 2019). In light of the predictive value of FH in both AD and depression, as well as the causal effect of depression on AD, based on genetic liability, we would like to examine how FH, which is linked with susceptibility, interacts with depression in explaining the severity of AD.

It should be noted that the etiology, course, and treatment of AD and depression differ among races and regions because of the disparities in genetics, social environment, and access to care for AD and depressive disorders. For example, in China, the prevalence of alcohol drinking problems is lower than in many Western populations, particularly among women (Im et al., 2019). This difference could reflect the variations in people's attitudes toward alcohol among different cultures. For example, social drinking is widely accepted among men but not among women in China. A systematic review found that, in China, pooled estimates of the current prevalence of AD, alcohol abuse, and AUD in men were 4.4, 4.0, and 10.1%, respectively, whereas the corresponding values for women were below 0.1, 0.1, and 0.2%, respectively (Cheng et al., 2015). Moreover, genetic factors may explain why the prevalence of problematic alcohol use in China is lower than in Western countries, as the unpleasant flushing response upon alcohol drinking due to a deficiency in metabolizing alcohol is common in Chinese populations. Therefore, as the Chinese population is underrepresented in studies of these disorders, it is vital to investigate alcohol dependence and its associated factors specifically in China, as the results of other studies may not be applicable or replicated explicitly in the Chinese background due to cultural differences.

Time perspective refers to how a person's subjective perception of the past, present, and future would influence their emotional, cognitive, motivational, and social processes, which, therefore, represents an important variable to deeply understand the relationship between depression and alcohol dependence. One of the most widely-used assessment measures of subjective time perspective is the Zimbardo Time Perspective Inventory (ZTPI) (Zimbardo and Boyd, 1999). Five dimensions have been outlined: (1) Future (F): engaging in the behavior to work steadily toward achievements; (2) Present hedonism (PH): taking pleasure in the present moment with little regard for the consequences; (3) Present fatalism (PF): believing that one's current efforts are useless and consequently disengaging from goal-oriented activities; (4) Past negative (PN): recalling negative or traumatic past experiences; and (5) Past positive (PP): recalling nostalgic and pleasant memories of the past. Despite abundant evidence suggesting that ZTPI has a stable structure and can be replicated in different countries and cultures, to understand fully the time perspective in the Chinese population, it is necessary to revise ZTPI and assess its psychometric properties in mainland China. Therefore, in our study, we used a Chinese short version of ZTPI (ZTPI-C), with its validity and reliability examined in other studies on various gender and age groups among the Chinese population (Li et al., 2022). The corresponding author who developed ZTPI-C pointed out that, according to his research (Wang and Lyu, 2016), the “present hedonistic” dimension could merely reflect the aspect of “impulsivity” in the revised version of ZTPI. Therefore, in ZTPI-C, they renamed the “Present Hedonistic” dimension as “Present Impulsivity,” which corresponds to the characteristics of impulsivity, carelessness, and disregard for consequences.

Time perspective plays a role in depressive disorders. In one study investigating adolescents in Hong Kong, researchers found that high depression levels were associated with higher negative past (PN) and fatalistic present (PF) and lower levels with positive past (PP), hedonistic present (PH), and future orientation (F) (Chan et al., 2019). In the development of ZTPI-C, authors reported significant correlations between depressive symptoms and PN (*r* = 0.45), PF (*r* = 0.29), and PP (*r* = –0.32) time perspectives among general populations in China, although PI and F showed insignificant results. Few studies have sought the relationship between time perspective and relation in the context of patients with psychiatric disorders. One study found that clinically depressed patients showed a tendency to focus more on negative past experiences (PN) and fatalism (F), whereas less on the present moment (PI) (Lefevre et al., 2019). In adult patients with attention deficit hyperactivity disorder (ADHD), depression was positively associated with PF and conversely associated with PP (Carelli and Wiberg, 2012).

Not only could motivation for alcohol use arise from environmental factors, such as family role models and the social environment where alcohol consumption is encouraged, but also personality factors, such as time perspective. More specifically, time perspective has been affirmed to be both a stable disposition and a transient attitude; and regression analyzes also indicate that time perspective is related to personality traits, yet can not be reduced to traits (Keough et al., 1999). It is generally accepted that a person's future perspective could help with maintaining illness preventive or healthy behaviors, while the present perspective might blind individuals from the potential harm and future risks that health-compromising behaviors could cause. Meanwhile, time orientation toward the past may influence the stress and tension, which are risk factors for increased unhealthy behaviors. Moreover, the relationship between time perspective and substance-related pursuit has been elucidated by multiple studies. For example, researchers found that, in Italian adolescents, the past positive perspective was associated with decreased binge drinking, while the opposite was found for past negative and present fatalistic time perspectives (Laghi et al., 2012). Another study also found past negative associated with greater alcohol and illicit drug use consequences, as some individuals might use substances as a way to cope with the negative affect often associated with the past negative time perspective (Chavarria et al., 2015) . Present time perspective, hedonism, and impulsivity have been related to heavier and more frequent alcohol consumption (Keough et al., 1999; Shin et al., 2012). As for future time perspective, it has been identified as a protective factor associated with decreased problematic alcohol use (Keough et al., 1999; Wagner et al., 2020). Although there is substantial evidence that addictive disorders are associated with time perspective, few studies have specifically investigated patients in the context of AUD or AD. One study indicated that future time perspective could affect the level of alcohol-related problems (Wagner et al., 2020), but the sample was small (*n* = 79). Another study from France enrolled outpatient participants (*n* = 139) and added all five dimensions into one regression model to predict the severity of AUD; however, no significant variable was found (Loose et al., 2018). Therefore, further investigations with larger samples are warranted to unravel the relationship between time perspectives and AD in patients with AD.

The literature suggests a relationship between time perspective and depression and alcohol dependence; however, the results of previous studies showed a discrepancy. The inconsistency of the results could result from differences in the participants (general population, students, patients with mental health problems, and individuals with other characteristics or issues.) The heterogeneity could also be caused by different questionnaires utilized in the study to assess depression and alcohol-related problems. More importantly, earlier studies have not focused on the moderating role of time perspective or FH, exploring when depression could predict AD severity. Thus, our study is the first to examine the moderation effect of time perspective and FH in the relationship between depression and AD.

### The aim of this study

The etiology of co-morbid depression and AD has been widely studied. In addition, many studies have addressed AD, depression, and how time perspective and FH are associated with the two disorders. However, neither the time perspective nor FH's role has been explicitly studied in the relationship between depression and AD in a clinical setting. Furthermore, data from Chinese cultural backgrounds are warranted, which would make valuable contributions to the existing body of research. This study aimed to examine the cross-sectional condition of the temporal profile, depression severity, and alcohol dependence in patients with AD in Chinese addiction treatment clinics. We also investigated how scores of the five ZTPI sub-scales and sociodemographic information would relate to the severity of depression and predict the severity of AD. Accordingly, the following hypothesis was proposed:

**Hypothesis 1 (H1)**: Among patients with alcohol dependence, the Zung Self-Rating Depression Scale (SDS) score, Past Negative score (PN), Present fatalistic score (PF), Present impulsive score (PI), as well as a positive family history of alcohol dependence (FH) will positively predict Michigan Alcoholism Screening Test (MAST) score; Past positive score (PP) and Future score (F) will negatively predict MAST. SDS score is positively correlated with PN and PF and negatively correlated with PI, F, and PP.

Few studies target patients with alcohol dependence, investigating the role depression plays in contributing to AD severity. In light of previous studies indicating genetic susceptibility could explain the causative effect of depression on AD (Polimanti et al., 2019), and FH could associate with genetic factors underlying depression and AD, we supposed that depression could predict the severity of AD, and FH and time perspective could moderate this relationship. In our study, we explored how time perspective and FH would interact with depression when depression acts as the predictor of AD severity. Hence, the following hypotheses were additionally proposed:

**Hypothesis 2 (H2)**: Among patients with AD, FH and time perspective (including PN, PI, PF, F, PP) would interact with depression and have a moderate association between depression and AD.

Our study is expected to expand the knowledge regarding the comorbidity of depression and alcohol dependence in patients with AD. It is of contemporary relevance as its results can help target interventions for patients experiencing or those susceptible to experiencing the disorders of alcohol use and depression as comorbidity. Moreover, it is especially relevant to the Chinese context, considering the genetic differences between Chinese and Western individuals and social differences related to alcohol consumption in Chinese context.

## Methods

### Participants

Our research interest was in Alcohol Dependent patients, which existed in DSM-4 and ICD-10 instead of DSM-5. AD is more severe and devastating compared to alcohol abuse, and previous research reveals that genetic susceptibility could explain the causative effect of depression on AD, instead of AUD. In our study design, we decided to choose AD patients diagnosed by psychiatrists, instead of both AD and alcohol abuse patients.

Questionnaires were distributed by trained healthcare workers in outpatient and inpatient departments of multiple psychiatric clinics, including psychiatric hospitals located in Wenzhou, Hangzhou, and Shaoxing, from January to December 2020. The inclusion criteria of participants were: (1) Diagnosed with AD according to the ICD-10; (2) having a stable mental condition, not accompanied by hallucinations, delusions, or other psychotic symptoms; having clear consciousness, and the ability to understand the questionnaire; (3) signing the informed consent or getting it signed by a family guardian; (4) being aged between 18 and 75 years old.

The exclusion criteria, based on the structural interview by medical staff, were the following: (1) having other psychiatric disorders such as intellectual disability, dementia, schizophrenia, and affective disorders; (2) having severe and unstable major somatic diseases; (3) engaging in abuse of other psychoactive substances.

We used G*Power software (Version 3.1.9.6, MacOS) (Faul et al., 2007) to calculate statistical power in our study. According to previous research in which the correlation coefficient was between ± 0.1 and ± 0.5 (Li et al., 2022), *R*^2^ was from 0.10 to 0.21, and Δ*R*^2^ was from 0.001 to 0.005 (Loose et al., 2018), we had estimated the effect size in correlation analysis as 0.4, the effect size of multiple linear regression (including hierarchical regression) as 0.02 (one-tailed), and therefore, the required minimum sample size for the power of 0.8 was 311. The study consisted of 401 patients (381 men, 20 women). 95.05% of participants were men, and only 4.95% were women, consistent with one systematic review (Cheng et al., 2015) that found that pooled estimates of the prevalence of AD in men were 20 times higher than in women (4.4 vs. 0.1% ). After eliminating cases with missing values in quantitative scales, questionnaires by 384 participants (365 men, 19 women) with valid scores in ZTPI, MAST, and SDS scales were shortlisted. Meanwhile, 39 participants skipped some sociodemographic questions. Therefore, different characteristics may have varying total numbers of cases when comparing means of quantitative variables (ZTPI, MAST, SDS scores) among subgroups. When adding all descriptive variables in the regression model, we considered only 345 participants who had answered all the questions (indicating a response rate of 86.03%).

### Measurement

#### Socio-demographic information

In our survey, participants were asked to report their gender, age, marital status, employment status (including retirement), education level, monthly income, and whether they lived alone or with others. They were also asked about who mainly took care of them (which meant whether they were being visited regularly and supervised to take medications), family history of AD (parents or grandparents), for how long they had been drinking heavily (consuming more than four drinks on any day for men and more than three drinks for women), and the number of times they had been to psychiatry clinics for alcohol use problems. We added demographic variables to the regression models to see whether these potential risk factors would impact the dependent variables.

#### Zimbardo time perspective inventory-Chinese version

Our study used a validated Chinese version of ZTPI (ZTPI-C). The author indicated that, after revision of the inventory, the “present hedonistic” dimension only reflected impulsivity. Therefore, similar to the change implemented by the authors, we also used “Present Impulsivity” instead of “Present Hedonistic,” (Wang and Lyu, 2016), which is associated with the characteristics of impulsivity, carelessness, and disregard for consequences. The ZTPI-C scale contains 25 items and five subscales: past-negative (PN, six items; e.g., “Painful past experiences keep replaying in my mind”), past-positive (PP, seven items; e.g., “In balance, there is much more good to recall than bad in my past”), present-impulsive (PI, four items; e.g., “I often follow my heart more than my head.”), present-fatalism (PF, three items; e.g., “My life path is controlled by forces I cannot influence.”), and future (F, five items; “Meeting tomorrow's deadlines and doing other necessary work comes before tonight's play.”). Participants were requested to rate how characteristic each item is of them on a 5-point Likert scale, ranging from 1 (“very uncharacteristic”) to 5 (“very characteristic”).

#### Zung self-rating depression scale

The SDS consists of 20 items with a 4-point Likert scale (Zung, 1965) and is used to screen adults for the potential presence of depressive disorders. Our study used a Chinese version of the SDS scale, which has been widely used and tested for its validity and reliability. In previous epidemiology investigations of SDS scores in China, people having a raw SDS score higher than 41 were considered to have depression (Wang et al., 1986; Dunstan and Scott, 2019). In our study, 59.90% of participants reached the threshold value of depression. We classified patients into "depression" and "not depression" and compared between two groups regarding MAST scores and time perspective scores.

#### Michigan alcoholism screening test

The MAST is a self-reported questionnaire developed to help detect AD (Selzer, 1971). The 24 items with weights from 0 to 5 tap various problems associated with alcohol use during the patient's lifetime. The total MAST score can range from 0 to 53 (Selzer et al., 1975). According to an investigation in China, using the weighted method, patients with a MAST score higher than 5 could be considered to have alcohol use problems (Yang, 2016). All patients in this study had a MAST score higher than 5 (Table 1).

**Table 1:** Age, times to alcoholism treatment center, MAST, and SDS scores in different demographic subgroups.

		**Age**		**Times**		**MAST**		**SDS**	
	**Number (%)**	**Mean (SD)**	**Effect**	**Mean (SD)**	**Effect**	**Mean (SD)**	**Effect**	**Mean (SD)**	**Effect**
**Gender**									
Male	365 (95.1)	49.0 (14.0)^a^	0.33	3.1 (4.3)	0.23	24.3 (8.2)	0.38	44.0 (15.0)	—0.46^*^
Female	19 (4.9)	43.0 (21.0)^a^		2.1 (1.9)		21.1 (10.5)		48.0 (10.0)	
**Medical treatment type**									
Inpatient	369 (96.1)	49.0 (14.0)^a^	0.46	3.0 (4.3)	0.33	24.1 (8.3)	0.06	24.1 (8.3)	0.13
Outpatient	15 (3.9)	45.0 (13.0)^a^		1.7 (0.7)		23.6 (7.9)		23.6 (7.9)	
**Insurance type**									
Free	39 (10.2)	49.2 (9.1)	0.01	2.2 (1.4)	0.01	24.9 (9.0)	0.00	37.0 (11.0)^a^	0.14^**^
Rural	101 (26.3)	48.2 (9.3)		3.3 (5.2)		24.2 (8.1)		42.0 (13.0)^a^	
Self-paid	67 (17.4)	46.6 (9.1)		2.5 (2.9)		23.5 (9.1)		51.0 (14.0)^a^	
Urban	170 (44.3)	48.0 (10.1)		3.2 (4.5)		24.3 (7.9)		47.0 (14.0)^a^	
Unreported	7(1.8)	48.9 (6.8)		3.1 (3.5)		19.9 (11.0)		44.0 (17.0)^a^	
**Are you employed now?**									
No	75 (19.5)	48.0 (12.0)^a^	–0.12	4.5 (7.0)	0.43^**^	24.9 (9.3)	0.14	46.2 (13.0)^a^	0.09
Yes	305 (79.4)	49.0 (15.0)^a^		2.7 (3.2)		24.0 (8.1)		44.2 (15.0)^a^	
Unreported	4 (1.0)	43.0 (16)^a^		3.7 (2.0)		22.8 (6.7)		52.5 (9.0)^a^	
**Education level**									
1	249 (65.1)	50.0 (8.8)	0.10^**^	2.7 (3.9)	0.02	23.2 (7.8)	0.02	44.2 (9.4)	0.01
2	69 (18.0)	45.4 (9.9)		4.1 (5.3)		25.3 (9.5)		44.5 (10.1)	
3	30 (7.8)	43.1 (10.0)		2.4 (1.4)		25.7 (7.9)		41.7 (10.5)	
4	31 (8.1)	41.6 (9.3)		2.8 (5.4)		26.2 (8.9)		43.9 (9.9)	
Unreported	4 (1.0)	52.0 (6.7)		5.2 (2.4)		32.5 (4.4)		41.5 (15.7)	
**Income**									
<2,000	115 (29.9)	51.0 (12.0)^a^	0.03^**^	4.2 (6.1)	0.04^**^	24.4 (8.4)	0.06^**^	46.1 (15.0)	0.05^**^
2,000–4,999	138 (35.9)	50.0 (14.0)^a^		2.3 (2.3)		21.6 (8.1)		46.4 (17.0)	
5,000–9,999	76 (19.8)	47.0 (15.0)^a^		3.2 (4.6)		26.6 (7.4)		44.8 (13.0)	
>10,000	52 (13.5)	43.0 (12.0)^a^		2.0 (1.7)		26.1 (8.5)		39.6 (13.0)	
Unreported	3 (0.8)	54.0 (0.0)^a^		5.0 (2.6)		33.7 (3.1)		55.7 (4.2)	
**Marrital status**									
Unmarried	51 (13.3)	43.7 (9.4)	0.07^**^	4.0 (5.8)	0.02	24.7 (8.9)	0.03^*^	47.3 (9.0)	0.03^*^
Married	258 (67.2)	48.7 (9.3)		2.7 (3.5)		23.7 (8.0)		44.0 (16.0)	
Divorced	62 (16.1)	46.6 (8.5)		2.8 (3.0)		26.4 (8.1)		43.2 (16.0)	
Widowed	12 (3.1)	57.5 (10.6)		5.3 (10.1)		19.3 (10.5)		46.3 (14.0)	
Unreported	1 (0.3)	60.0		7.0		14.0		32.0	
**Do you live alone?**									
Yes	79 (20.6)	48.8 (8.2)	0.10	2.9 (3.2)	–0.02	25.7 (8.9)	0.25^*^	43.3 (8.0)	—0.09
No	303 (78.9)	47.8 (9.9)		3.0 (4.4)		23.7 (8.2)		44.1 (10.1)	
Unreported	2 (0.5)	44.0 (9.9)		1.5 (0.7)		21.5 (5.0)		56.5 (3.5)	
**Who is taking care of you?**									
Spouse	220 (57.3)	49.5 (9.3)	0.13^**^	2.8 (4.2)	0.01	23.3 (8.1)	0.02	44.5 (16.0)^a^	0.00
Parents	69 (18.0)	41.2 (8.0)		3.4 (4.8)		26.4 (8.8)		45.0 (12.0)^a^	
Children	33 (8.6)	52.4 (9.3)		2.7 (2.3)		24.0 (7.2)		44.0 (15.0)^a^	
Others	60 (15.6)	47.5 (8.7)		3.5 (4.1)		24.5 (9.1)		44.0 (13.0)^a^	
Unreported	2 (0.5)	53.0 (4.2)		1		28.0 (4.2)		54.5^a^	
**Family AD history**									
No	157 (40.9)	49.0 (14.0)^a^	0.13	3.4 (5.6)	0.15	23.6 (8.1)	—0.10	49.0 (17.0)^a^	0.44^**^
Yes	226 (58.9)	48.5 (13.0)^a^		2.7 (2.9)		24.5 (8.5)		43.0 (14.0)^a^	
Unreported	1 (0.3)	39.0^a^		3.0		19.0		59.0^a^	
**Years of heavy drinking**									
<1 year	6 (1.6)	45.8 (16.9)	0.11^**^	3.3 (3.3)	0.00	21.3 (8.1)	0.01	20.5 (15.0)^a^	0.04^**^
1–5 years	18 (4.7)	39.9 (14.3)		3.1 (4.1)		23.3 (8.8)		25.0 (12.0)^a^	
5–10 years	23 (6.0)	38.3 (14.3)		2.1 (1.5)		26.2 (7.6)		27.0 (8.0)^a^	
>10 years	337 (87.8)	49.1 (8.5)		3.1 (4.4)		24.1 (8.4)		24.0 (15.0)^a^	
**Depression?**									
No	154 (40.1)	38.8 (8.5)^a^	0.14	2.8 (4.2)	–0.07	22.4 (8.0)	–0.34^**^		
Yes	230 (59.9)	47.4 (10.0)^a^		3.1 (4.2)		25.2 (8.3)			

a Data described as Median ± Q(r), default as Mean ± SD;

* *p* < 0.05;

** *p* < 0.01 (two-tailed).

### Reliability of questionnaires

The Cronbach's alpha of each scale was as follows: 0.837 for the SDS scale, 0.708 for the PN dimension of ZTPI, 0.749 for the PP dimension of ZTPI, 0.867 for the future dimension of ZTPI, 0.661 for the PI dimension of ZTPI, and 0.671 for the present fatalistic dimension of ZTPI. For the MAST scale, we took the standardized Cronbach's alpha because, on the MAST scale, the items did not have the same metric. Some dichotomous items scored 5, some scored 2, and some had 1. Cronbach's alpha based on standardized items or MAST was 0.718.

The Cronbach's alpha values of PI and PF scales were lower than 0.7, typically considered the threshold for the reliability test. Nevertheless, these results were in line with the initial study developing ZPTI-C (Li et al., 2022), in which Cronbach's alpha values of PI and PF were 0.70 and 0.57, respectively. This finding could be attributed to the small number of questions, with PI having 4 items and PF having 3 items only, and are considered to be adequate for measuring psychological constructs (Niemand and Mai, 2018).

### Statistical analysis

Data were analyzed with the SPSS Version 27.0 (IBM SPSS Statistics 27). All the mean comparison tests were two-tailed, and *p* < 0.05 was considered statistically significant. Due to the hypothesis-generating nature of these analyzes, no correction for multiple tests was performed.

#### Descriptive statistics and compare means

According to the Central Limit Theorem, when the sample is large enough (usually *n* ≥ 30), the distribution of sample means will be approximately normally distributed. We, therefore, applied the normal probability model to quantify uncertainty when making inferences about a population mean based on the sample mean.

For groups with data distributing normality, we used Mean ± SD for description, and for data without normality, we used Median ± Interquartile for description. To compare quantitative data between subgroups, we used *t*-tests or analysis of variance (ANOVA). We also calculated Cohen's d for the *t*-test and eta squared for ANOVA as effect size.

#### Pearson correlation

Correlation analysis was used to examine the extent to which two quantitative variables are linearly related. For quantitative and ordinal variables, we used Pearson's correlation. These analyzes quantify the direction and strength of the relationship between two variables.

#### Multiple linear regression

For multiple linear regression, the following few assumptions needed to be tested: (i) each predictor has a linear relation with our outcome variable; (ii) the prediction errors are normally distributed in the population; (iii) the variance of the errors is constant in the population (homoscedasticity). Assumptions were checked before making conclusions.

Michigan Alcoholism Screening Test was the outcome value. Besides the scores for five dimensions of time perspective (PN, PP, F, PI, and PF), we also input age and times (times to the AUD treatment clinic) into the regression model. “Income,” “education level,” and “years of heavy drinking” were ordinal variables, and we put the directional numbers into the model. We had dummy-coded nominal variables into multiple dichotomous variables, including insurance type, marital status, family history, caretaker, gender, inpatient or outpatient treatment, and whether the participant lived alone. This step was followed by adding these variables into the regression model. We eliminated dummy-coded marital status from the regressors because the variance inflation factor went above 5 when adding this variable.

Stepwise linear regression analysis was used to identify possible predictors of the outcome out of the above-listed candidate variables. At each step, variables were added based on *p*-values and the criteria in which probability-of-F-to-enter. ≤ 0.05, Probability-of-F-to-remove ≥ 0.1, was used to limit the total number of variables included in the final model. Adjusted R square indicated the proportion of variance in the dependent variable accounted for by the predicted values. Standardized coefficients *β* were the values for the regression equation for predicting the dependent variable from the independent variable after standardization.

#### Moderation analysis

To conduct moderation analysis, we utilized hierarchical multiple regression analysis and bootstrap analysis employing the PROCESS macro (model 1, 2) (Hayes, 2017). We first standardized variables and then added z-scored variables into regression models. Further, indirect effects were estimated using 5,000 bootstrapped resamples at 95% confidence intervals (CIs). When 95% CI did not include zero, the indirect effects were considered significant. All variables were centered.

## Results

### Sociodemographic characteristics

The composition of participants and values of quantitative variables in demographic subgroups in the study are presented in Tables 1, 2. For overall mean ± SD in PN, PP, F, PI, and PF, the scores were 19.16 ± 4.40, 24.30 ± 4.99, 16.57 ± 4.82, 13.05 ± 3.27, and 9.83 ± 2.64, respectively.

**Table 2:** ZTPI 5 dimension scores in different demographic subgroups.

		**PN**		**PP**		**F**		**PI**		**PF**	
	**Number (%)**	**M(IQR)**	**Effect**	**M(IQR)**	**Effect**	**M(IQR)**	**Effect**	**M(IQR)**	**Effect**	**M(IQR)**	**Effect**
**Gender**											
Male	365 (95.1)	19.0 (6.0)	—0.39^*^	25.0 (6.0)	0.14	17.0 (7.0)	–0.29	13.0 (5.0)	0.19	10.0 (4.0)	—0.15
Female	19 (4.9)	22.0 (4.0)		26.0 (10.0)		20.0 (8.0)		13.0 (4.0)		10.0 (3.0)	
**Medical treatment type**											
Inpatient	369 (96.1)	19.2 (4.4)^a^	0.42	24.3 (5.0)	–0.24	17.0 (7.0)	—0.34	13.0 (5.0)	0.64^*^	10.0 (4.0)	0.41
Outpatient	15 (3.9)	17.4 (5.3)^a^		25.5 (4.2)		18.0 (4.0)		12.0 (6.0)		10.0 (6.0)	
**Insurance type**											
Free	39 (10.2)	19.4 (4.5)^a^	0.02	26.0 (8.0)	0.03^*^	19.0 (6.0)	0.11^*^	11.0 (4.0)	0.07^**^	9.0 (4.0)	0.05^**^
Rural	101 (26.3)	19.6 (4.5)^a^		25.0 (6.0)		18.0 (5.0)		13.0 (5.0)		10.0 (4.0)	
Self-paid	67 (17.4)	18.1 (4.1)^a^		24.0 (6.0)		15.0 (10.0)		15.0 (4.0)		11.0 (3.0)	
Urban	170 (44.3)	19.2 (4.3)^a^		25.0 (8.0)		17.0 (7.0)		14.0 (5.0)		11.0 (3.0)	
Unreported	7(1.8)	20.6 (5.9)^a^		25.0 (5.0)		17.0 (6.0)		13.0 (8.0)		10.0 (7.0)	
**Are you employed now?**											
No	75 (19.5)	19.0 (4.2)^a^	–0.04	24.0 (7.0)	–0.24	18.0 (5.0)	–0.02	14.0 (4.0)	0.26	10.0 (3.0)	0.19
Yes	305 (79.4)	19.2 (4.5)^a^		25.0 (6.0)		17.0 (7.0)		13.0 (5.0)		10.0 (4.0)	
Unreported	4 (1.0)	18.8 (4.2)^a^		26.0 (10.0)		14.5 (9.0)		13.5 (7.0)		12.0 (4.0)	
**Education level**											
1	249 (65.1)	19.0 (5.0)	0.02	25.0 (6.0)	0.03^*^	17.0 (7.0)	0.04^**^	13.0 (5.0)	0.01	11.0 (3.0)	0.04^**^
2	69 (18.0)	20.0 (5.0)		26.0 (5.0)		18.0 (6.0)		13.0 (4.0)		10.0 (4.0)	
3	30 (7.8)	19.0 (6.0)		27.0 (6.0)		19.0 (7.0)		11.0 (5.0)		9.0 (3.0)	
4	31 (8.1)	19.0 (5.0)		25.0 (5.0)		19.0 (5.0)		13.0 (2.0)		9.0 (5.0)	
Unreported	4 (1.0)	25.0 (11.0)		25.0 (6.0)		18.0 (7.0)		14.0 (9.0)		10.0 (8.0)	
**Income**											
<2,000	115 (29.9)	18.8 (4.1)^a^	0.03^*^	24.0 (8.0)	0.04^*^	16.0 (7.0)	0.09^**^	14.0 (5.0)	0.02^*^	11.0 (3.0)	0.10^**^
2,000–4,999	138 (35.9)	18.6 (4.2)^a^		25.0 (6.0)		17.0 (9.0)		13.0 (4.0)		10.0 (4.0)	
5,000–9,999	76 (19.8)	20.6 (4.2)^a^		25.5 (3.0)		18.0 (4.0)		14.0 (4.0)		10.0 (3.0)	
>10,000	52 (13.5)	19.0 (5.1)^a^		26.0 (7.0)		20.0 (4.0)		12.0 (7.0)		8.0 (4.0)	
Unreported	3 (0.8)	26.7 (4.9)^a^		26.0		6.0		19.0		15.0	
**Marrital status**											
Unmarried	51 (13.3)	18.7 (4.3)^a^	0.01	23.0 (6.0)	0.04^**^	15.0 (8.0)	0.03^*^	14.0 (3.0)	0.02	12.0 (2.0)	0.05^**^
Married	258 (67.2)	19.1 (4.5)^a^		25.0 (6.0)		18.0 (6.0)		13.0 (5.0)		10.0 (3.0)	
Divorced	62 (16.1)	19.7 (4.1)^a^		26.0 (4.0)		18.0 (6.0)		13.5 (6.0)		10.5 (4.0)	
Widowed	12 (3.1)	19.6 (4.7)^a^		26.0 (5.0)		17.5 (8.0)		12.4 (4.0)		11.5 (4.0)	
Unreported	1 (0.3)	14.0^a^		19.0		15.0		11.0		6.0	
**Do you live alone?**											
Yes	79 (20.6)	19.9 (4.6)^a^	0.21	26.0 (6.0)	0.22	17.0 (5.0)	–0.01	13.5 (3.5)	0.18	11.0 (3.0)	0.27^*^
No	303 (78.9)	19.0 (4.3)^a^		25.0 (6.0)		17 0 (7.0)		12.9 (3.2)		10.0 (4.0)	
Unreported	2 (0.5)	15.0 (1.4)^a^		17.0		8.5		15.0			
**Who is taking care of you?**											
Spouse	220 (57.3)	19.0 (4.4)^a^	0.01	25.0 (5.0)	0.00	18.0 (6.0)	0.03^*^	13.0 (5.0)	0.04^**^	10.0 (3.0)	0.03^**^
Parents	69 (18.0)	19.7 (4.7)^a^		25.0 (6.0)		18.0 (5.0)		14.0 (6.0)		11.0 (3.0)	
Children	33 (8.6)	20.1 (4.0)^a^		26.0 (9.0)		16.0 (7.0)		12.0 (5.0)		10.0 (3.0)	
Others	60 (15.6)	18.8 (4.5)^a^		25.0 (8.0)		16.0 (9.0)		14.0 (4.0)		11.0 (3.0)	
Unreported	2 (0.5)	18.0 (2.8)^a^		23.0		13.5		13.0		12.0	
**Family AD history**											
No	157 (40.9)	18.6 (4.3)^a^	–0.23^*^	25.0 (7.0)	–0.28^**^	17.0 (8.0)	–0.38^**^	14.0 (5.0)	0.20^*^	10.0 (3.0)	0.17
Yes	226 (58.9)	19.6 (4.5)^a^		26.0 (6.0)		18.0 (6.0)		13.0 (5.0)		10.0 (4.0)	
Unreported	1 (0.3)	16.0^a^		22.0		7.0		16.0		12.0	
**Years of heavy drinking**											
<1 year	6 (1.6)	20.5 (2.2)^a^	0.00	26.5 (6.0)	0.01	14.5 (7.0)	0.00	15.0 (6.0)	0.00	11.0 (2.0)	0.01
1–5 years	18 (4.7)	19.5 (4.3)^a^		25.0 (8.0)		17.0 (6.0)		13.0 (4.0)		9.5 (4.0)	
5–10 years	23 (6.0)	19.8 (5.4)^a^		27.0 (7.0)		18.0 (10.0)		13.0 (7.0)		10.0 (6.0)	
>10 years	337 (87.8)	19.1 (4.4)^a^		25.0 (6.0)		17.0 (7.0)		13.0 (4.0)		10.0 (4.0)	
**Depression?**											
No	154 (40.1)	18.7 (4.2)^a^	–0.17	26.0 (6.0)	0.41^**^	19.2 (3.2)^a^	1.00^**^	11.8 (3.2)^a^	–0.69^**^	9.0 (3.0)	—0.92^**^
Yes	230 (59.9)	19.4 (4.5)^a^		24.5 (7.0)		14.8 (4.9)^a^		13.9 (3.0)^a^		11.0 (3.0)	

PN, past negative; PP, past positive; F, future; PI, present impulsive; PF, present fatalistic.

a Data described as Mean ± SD, default as Median ± Q(r);

* *p* < 0.05;

** *p* < 0.01 (two-tailed).

Patients being female, have a self-paid insurance type, negative family history of alcohol dependence, a longer duration of drinking, and lower income, tend to have a higher score of SDS. Medical treatment type (inpatient or outpatient) did not affect MAST or SDS. We also found that patients being visited regularly and supervised by parents tended to have higher SDS and MAST scores, and patients supervised by their spouse tended to have higher depression scores compared with having any other type of caregiver (such as friends or siblings) or no caregiver.

### Pearson's correlation coefficient

Table 3 demonstrates the correlation coefficient and the statistical significance between every pair of continuous variables. We found that most correlations were significant, with weak to moderate associations. Notably, we found moderate correlation within some time perspective dimensions, with F and PP having a correlation coefficient of 0.48 and PF and PI having a correlation coefficient of 0.51. PI and PF displayed a positive and significant correlation with both MAST (*r* = 0.23, *r* = 0.14, respectively) and SDS (*r* = 0.34, *r* = 0.44, respectively). PP and F displayed a negative correlation with SDS (*r* = –0.24, *r* = –0.55, respectively), yet showed no significant correlation with MAST (*r* = 0.08, *r* = –0.03, respectively). PN had a positive correlation with MAST (*r* = 0.23), and no significant correlation with SDS (*r* = 0.04). MAST and SDS were positively correlated.

**Table 3:** Correlation table.

	**Times**	**PN**	**PP**	**F**	**PI**	**PF**	**MAST**	**SDS**
Age	0.14^**^	–0.1	–0.1	–0.14^**^	–0.06	0.04	–0.08	–0.02
Times	1	0.12^*^	0.07	–0.02	0.08	0.02	0.24^**^	–0.01
PN	0.12^*^	1	0.31^**^	0.21^**^	0.32^**^	0.27^**^	0.23^**^	0.04
PP	0.07	0.31^**^	1	0.48^**^	–0.11^*^	–0.11^*^	0.08	–0.24^**^
F	–0.02	0.21^**^	0.48^**^	1	–0.38^**^	–0.38^**^	–0.03	–0.55^**^
PI	0.08	0.32^**^	–0.11^*^	–0.38^**^	1	0.51^**^	0.23^**^	0.34^**^
PF	0.02	0.27^**^	–0.11^*^	–0.38^**^	0.51^**^	1	0.14^**^	0.44^**^
MAST	0.24^**^	0.23^**^	0.08	–0.03	0.23^**^	0.14^**^	1	0.11^*^

PN: past negative; PP: past positive; F: future; PI: present impulsive.

PF: present fatalistic; times: times to alcoholism treatment center.

* *p* < 0.05;

** *p* < 0.01.

### Predicting factors of MAST

We performed linear regression analysis to investigate further which variables could predict the MAST score. The stepwise regression model started with 22 variables that might potentially predict MAST, and then a forward stepwise linear regression model was applied to reduce them to seven significant predictors, which were: the times that the patient went to an AUD treatment center, PN, PI, income, caretaker being parent, SDS, and living alone (*R*^2^ = 0.17, *P* < 0.01). Higher scores in PN (*β* = 0.13, *p* = 0.01), PI (*β* = 0.12, *p* = 0.03), and the SDS score (*β* = 0.12, *p* = 0.03) positively predicted the MAST score. Patients with more frequent relapse (*β* = 0.24, *p* < 0.01) and higher income (*β* = 0.18, *p* < 0.01) had more severe AD. We also found that patients who lived alone (*β* = 0.10, *p* = 0.05), and were supervised and visited by parents to have their condition monitored (*β* = 0.12, *p* = 0.01) tended to have higher MAST scores (Table 4).

**Table 4:** Stepwise linear regression model predicting MAST score.

**Model summary**			**Coefficient**				
**R**	**R^2^**	**Adjusted R^2^**	**Variable**	***β***	***t***	**LCI**	**UCI**
0.41	0.17	0.15	(Constant)		1.69	–0.82	10.93
			Times	0.24	4.70^**^	0.27	0.66
			PN	0.13	2.52^*^	0.06	0.45
			PI	0.12	2.13^*^	0.02	0.58
			Income	0.18	3.53^**^	0.67	2.35
			Caretaker2	0.12	2.48^*^	0.57	4.87
			SDS	0.12	2.20^*^	0.01	0.19
			Live alone	0.10	1.98^*^	0.02	4.25

PN, past negative; PI, present impulsive; Times, times to alcoholism treatment center; Caretaker2, parents.

* *p* < 0.05;

** *p* < 0.01.

We also constructed a regression model including all 22 variables. In this condition, we would see whether sociodemographic characteristics would impact other predictors. In the model predicting MAST using the "enter" method, only three variables were significant (Table 5), four less than in the "stepwise" method. PN, PI, living alone, and supervision by parents were no longer significant in the model. Did this mean that time perspective was not associated with AD when adjusted for confounding sociodemographic factors? The *p*-values for PN and PI were both 0.08, very close to the arbitrary 0.05 threshold. Besides, the effect size, also known as standardized coefficient *β*, was 0.11 for both PN and PI, still close to 0.13 and 0.12 in the stepwise model. This phenomenon, in which the predictor became insignificant after adding more variables, did not negate its contribution to the dependent variable, especially when the object was not a large-scale study. According to the theory and process of regression, when adding more independent variables, the df increases, thus decreasing the F and altering *p*-values. Therefore, the *p*-value would shift between significant and insignificant when adding or removing variables. But we can still look at its effect size to see the strength of the predictor.

**Table 5:** Regression model of MAST adjusting for all demographic variables.

**Model summary**	**R^2^**	**F Change**	**Sig. F Change**
	0.20	3.71	< 0.01
**Coefficients**	***β***	**t**	**Sig**.
(Constant)		–0.17	0.86
Age	–0.07	-1.08	0.28
Income	0.16	2.54^*^	0.01
Education	0.10	1.62	0.11
Times	0.24	4.45^**^	< 0.01
Total	0.06	1.06	0.29
PN	0.11	1.78^$^	0.08
PP	0.02	0.35	0.73
F	–0.04	–0.56	0.58
PI	0.11	1.76^$^	0.08
PF	0.03	0.49	0.62
SDS	0.15	2.24^*^	0.03
Male	0.07	1.23	0.22
Inpatient	< 0.01	0.08	0.94
Live alone	0.08	1.18	0.24
Insurance3	0.08	1.14	0.25
Insurance2	0.03	0.46	0.65
Insurance1	0.10	1.48	0.14
FH+	0.06	1.04	0.30
No work	0.04	0.75	0.45
Caretaker1	–0.01	–0.13	0.90
Caretaker3	0.03	0.46	0.65
Caretaker2	0.08	0.99	0.32

$ *P* < 0.1;

* *P* < 0.05;

** *P* < 0.01. F, future perspective; PF, present fatalistic; PN, past negative; PI, present impulsive; PP, past positive; Times, times to alcoholism treatment center; Total= total years for heavy drinking; FH+, positive family history; Insurance1, free insurance; Insurance2, urban insurance; Insurance3, rural insurance; Caretaker1, spouse; Caretaker2, parents; Caretaker3, children.

### Moderation effect of time perspective and family history

To test the hypothesis that time perspective and FH moderate the predictive relationship of depression on AD, we performed hierarchical multiple regression as well as Hayes's process. All variables had been standardized. We conducted five moderation analyzes for each dimension of time perspective (Table 6), in which we selected model 2 in *PROCESS* macro and set each type of time perspective and family history as moderators. It turned out that, among the 5 dimensions of time perspective, only PN significantly moderated the effect of SDS on MAST, while family history had been consistently significant in the moderation models. Although no significant difference was detected in MAST scores between patients with or without a family history (24.5 ± 8.5 vs. 23.6 ± 8.1), family history still played a significant role in AD pathology.

**Table 6:** The results of analyzing the moderation effect of time perspective and family history on SDS and MAST.

**Models**	**R^2^**	**Interaction variable**	***β***	**SE**	***t***	***p***	**CI 95%**	
Past negative	0.09	PN*SDS	0.10	0.05	2.05	0.04	<0.01	0.2
		FH*SDS	0.12	0.05	2.37	0.02	0.02	0.22
Present impulsive	0.08	PI*SDS	0.07	0.05	1.43	0.15	–0.03	0.17
		FH*SDS	0.12	0.05	2.42	0.02	0.02	0.22
Past positive	0.05	PP*SDS	0.05	0.05	0.98	0.33	–0.05	0.15
		FH*SDS	0.13	0.05	2.54	0.01	0.03	0.23
Future	0.04	F*SDS	0.04	0.06	0.65	0.51	–0.07	0.15
		FH*SDS	0.13	0.05	2.41	0.02	0.02	0.23
Present fatalistic	0.05	PF*SDS	0.05	0.05	1.05	0.30	–0.05	0.15
		FH*SDS	0.13	0.05	2.59	0.01	0.03	0.23

In addition, since a family history of AD could lead to higher scores of PN than no history, we tested whether the interaction between PN and FH would also significantly influence the association between depression and AD. After putting SDS, PN, FH, and the interaction terms, PN*SDS, FH*SDS PN*FH into the hierarchical regression model, the interaction between PN*FH was insignificant in predicting MAST (*β* = –0.03, *p* = 0.5). Therefore, PN did not interact with FH, while they both interact with SDS.

**Figure 1:**
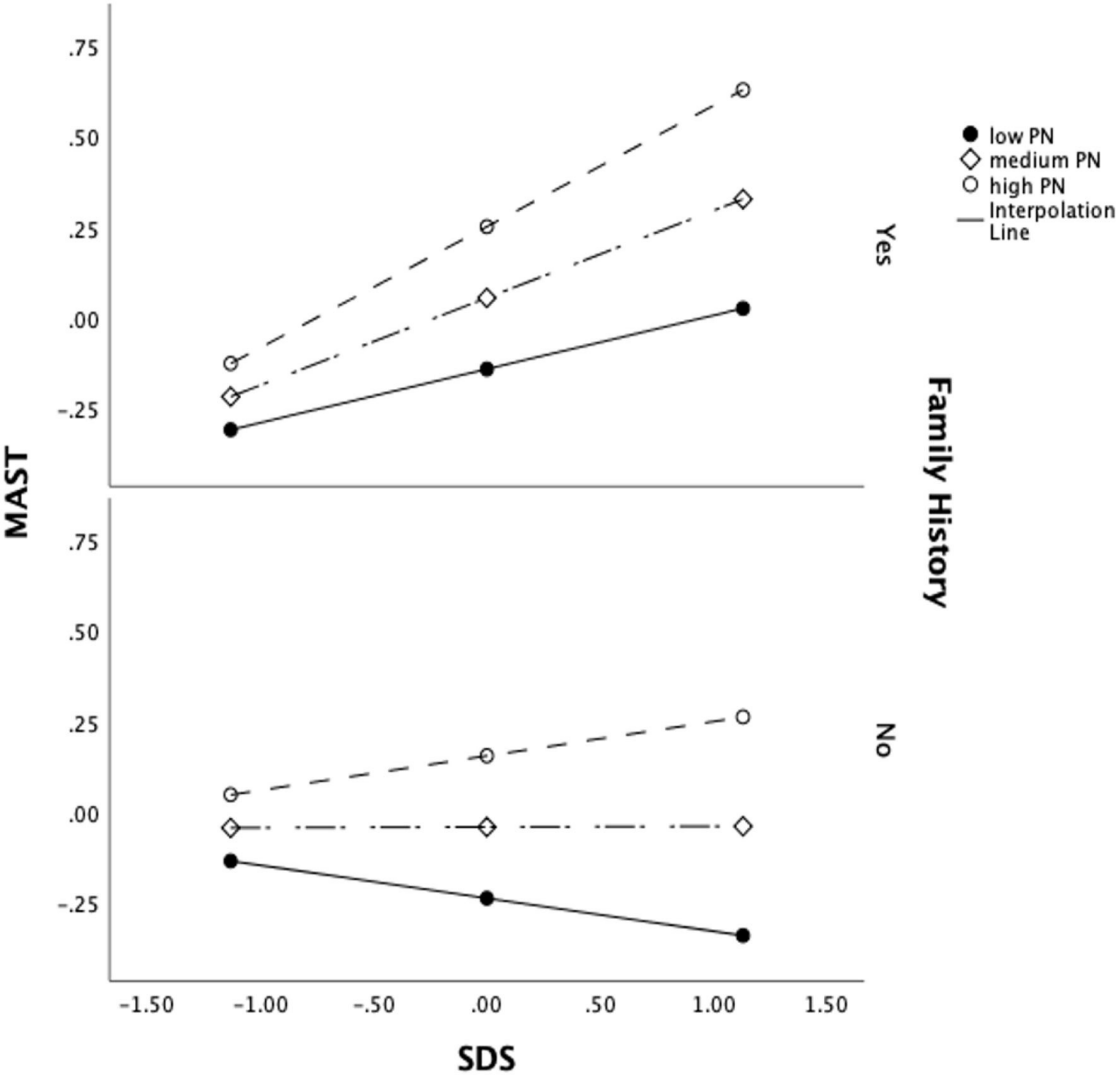
Moderation interaction plot.

**Figure 2:**
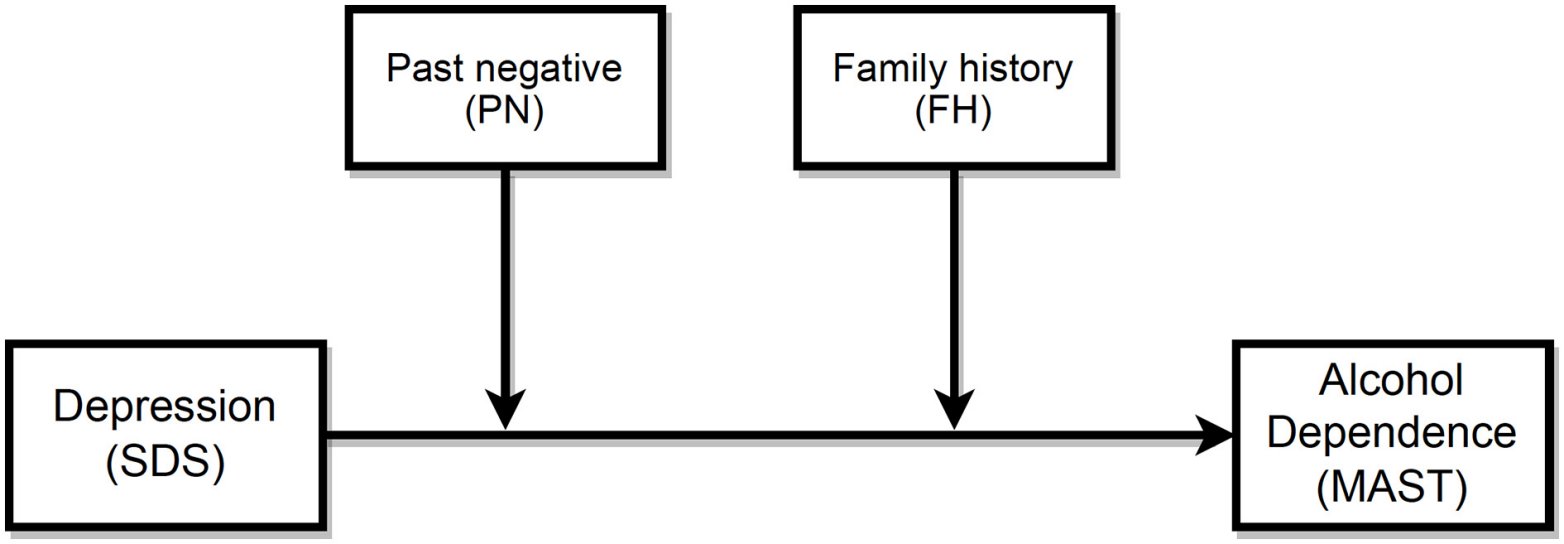
Moderation model.

The results of conditional effects of the SDS as the predictor of MAST, with PN and FH being the moderators, are shown in Table 7. To depict the moderation effect more vividly, we also generated the interaction plot (Figure 1), which showed an enhancing effect of moderators, i.e., as the PN score increased, the MAST score increased. At a low PN score, patients' MAST scores were similar among patients with low, average, or high SDS scores. However, at average and high levels of PN score, patients' SDS scores positively predicted MAST. Interestingly, SDS only had a strong relationship with MAST in patients with a family history of alcohol dependence, indicating that a negative FH would act as a protective factor between depression and AD. The overall moderation effect model can be referred to in Figure 2.

**Table 7:** Conditional effects of SDS predicting MAST at different values of the moderators.

**Past negative**	**Family history**	**Effect**	**SE**	***p***	**LLCI**	**ULCI**
Low	No	–0.08	0.07	0.26	–0.22	0.06
Average	No	0	0.06	0.98	–0.12	0.12
High	No	0.08	0.08	0.28	–0.07	0.23
Low	Yes	0.13	0.07	0.08	–0.02	0.27
Average	Yes	0.21	0.06	<0.01	0.09	0.33
High	Yes	0.29	0.07	<0.01	0.15	0.43

## Discussion

The main goal of this cross-sectional study was to investigate the effect of time perspective and FH on the association between depression and AD. This study had 384 samples from multiple clinical centers, making our results more representative and solid. In addition, in the Chinese cultural context, our cross-sectional study could contribute to the fields of time perspective, alcohol dependence, and its comorbidity with depression. As data in these fields is relatively insufficient regarding the Chinese population, the current study shall fill the gap and expand the related dataset.

This study was progressive from simple to complex. We first demonstrated demographic characteristics and used a *t*-test or ANOVA. Then the correlation matrix would provide information about the quantitative variables. We then performed linear regression analyzes and moderation analyzes to examine the role time perspective and FH play in predicting MAST. Taken together, our findings demonstrate that time orientation is an important construct in individual psychological functioning, as it is related to the severity of depression and alcohol dependence in patients with AD. More importantly, we are the first to find that time perspective and FH moderate the effect of depression on AD.

For the demographic factors, male gender, FH (family AD history of first or second degree relative), and longer duration of heavy drinking were found to be associated with less depression. Consistent with previous research (Dawson and Grant, 1998), the male gender had a negative association with depression. Although many studies have shown that FH is positively associated with depression, alcohol dependence, and comorbidity (Hasegawa et al., 1991; Dawson and Grant, 1998), our results suggest otherwise. Similarly, this discrepancy might be due to the different research contexts. For example, one study indicated that familial alcoholism would contribute to depression. In this study, researchers collected data from a nationwide epidemiologic survey and drew conclusions by comparing the prevalence between people with or without psychiatric disorders (Dawson and Grant, 1998). Analyzing binary data (depression or not, alcoholism or not), and performing logistic regression, they found significantly elevated odds ratios for depression and alcoholism in patients with family histories of alcohol dependence. However, in our study, all participants had been diagnosed with AD, and as mentioned above, their time perspectives and psychological mechanisms were altered by chronic psychiatric disorders. We explored the relationship between variables within the context of these patients' situations. Therefore, the results from other studies likely do not apply. Interestingly, we found one study that exclusively investigated AD patients (*n* = 70). In it, patients with a positive FH had a lower major depression disorder rate (25.4%) than patients with a negative FH (63.6%), which was consistent with our study results (Abraham et al., 2018).

Correlation analysis indicated that PN, PI, and PF were positively correlated with MAST scores, consistent with findings that past negative (Chavarria et al., 2015) and present time perspective (Keough et al., 1999; Chavarria et al., 2015), including present hedonism (PI in our study) and present fatalism, were significant predictors of substance use. While an orientation toward the present could aggravate alcohol dependence through impulsive reward-seeking, an orientation toward the negative past would exacerbate alcohol misuse through increasing stress or depressive feelings, changes that consequently lead to excessive alcohol consumption. However, contradicting most studies showing PN being significantly positively correlated with SDS, in our study (Table 3), PN had no significant correlation with SDS, with a very low coefficient (*r* = 0.04). Studies have shown that depressed patients showed decreased present hedonism (PH) scores (Zimbardo and Boyd, 1999; Lefevre et al., 2019). Depressed individuals generally experience a lack of hedonism, which means difficulty in experiencing pleasure and interest in activities. On the contrary, the result about present impulsivity (PI) in our study indicated otherwise, with a positive correlation with SDS (*r* = 0.34). This converse finding might stem from the property of ZTPI-C's PI subscale, which, as the authors indicated, merely reflected impulsiveness, instead of hedonism (Li et al., 2022). Research has also indicated that, in alcohol-dependent patients, impulsivity is the strongest predictor of depression severity, as shown in linear regression models (Jakubczyk et al., 2012). Similarly, another study specifically investigated individuals seeking help for alcohol and drug dependence issues and found that PH was significantly positively associated with depression (Davies and Filippopoulos, 2015). These findings align with our study.

The regression models in our study also support the idea that time perspective can predict the severity of AD in patients with AD. Meanwhile, we also adjusted confounding demographic factors and found that having more rounds of detoxication therapy in psychiatric clinics, higher income, and living alone could predict higher scores of MAST. Research supports the finding that alcohol misuse is correlated with less advantaged living arrangements (Joutsenniemi et al., 2007). Living alone would increase feelings of loneliness, which are linked to addiction and AD. Studies investigating the relationship between socioeconomic conditions and alcohol use disorders yielded mixed results. One study indicated that genetics play a more substantial role in the drinking habits of people with low incomes. In contrast, environmental factors were more influential for people with higher incomes (Hamdi et al., 2015). Additionally, in our study, FH did not predict MAST (*β* = 0.06, *p* = 0.3), but acted as a protective factor against depression. The unexpected results related to FH and heavy drinking duration may relate to alcoholism type. Type II alcoholism, being primarily genetically driven, has an earlier age of onset. Hence, it is more closely associated with a family history of alcohol-related problems. Type I alcoholism, being less genetic in nature, has a later age of onset but is more closely related to anxiety and depression (Cloninger et al., 1996). This difference may explain the negative correlation between depression, which is related to Type I alcoholism, and FH and duration of alcohol abuse, which are related to Type II alcoholism's early onset.

Using moderation analysis, we found that, in patients with FH having average to high PN temporal profiles, depression level exacerbated their AD. A family history of alcohol dependence, as well as a deviated negative perception toward the past, would interact with depression and escalate its effect when predicting the severity of AD. Nevertheless, our moderation model explained the 9% variability of MAST (Table 5). MAST scores in our study had great variance. We included only five regressors in the model, which may account for this result. Moreover, it is hard to construct complete, well-specified models for AD severity, which is influenced by complicated factors and varies from person to person. Significant but low *R*^2^ is common in psychological studies. We noticed that in some studies, researchers had implemented regression analyzes to predict the time perspective effects on AUDIT (Alcohol use disorders identification test) scores, widely used to assess problematic alcohol consumption. In these studies, *R*^2^ was 14.2% (McKay et al., 2018) and 10.0% (Loose et al., 2018), respectively. Even though *R*^2^ was small, it revealed a significant contribution to the model. Meanwhile, one study that had included 325 variables in 69,187 participants, and implemented deep learning to predict outcome variables (Kim et al., 2021). This result suggests that the use of a larger dataset, the inclusion of more predictors, and the implementation of more advanced algorithms will be valuable in future studies.

### Implication for clinical practices

This cross-sectional study provides valuable understanding and knowledge for healthcare professionals working in specialized addiction treatment centers. Our results will support the design and delivery of interventions to treat patients and promote their healthy behaviors. Clinical healthcare workers in China should take note of the high comorbidity rates of AD and depression among AD patients. Moreover, this positive correlation was significant only in patients with a positive FH and average to high PN time perspective. Therefore, prior to the start of therapy, clinicians should screen for a family history of FH, determine the individual's time perspective and identify problem areas.

Consistent with previous studies, patients in AUD treatment clinics may exhibit high levels of regret and negativity toward the past and anxieties about what might happen in the future (Davies and Filippopoulos, 2015). Our findings suggest that addressing an individual's negative view of the past, particularly those with concurrent depressive symptoms, could help reduce AD severity, thereby facilitating successful alcohol detoxification. Furthermore, interventions should also promote a future time perspective while reducing PF time perspective, as they are associated with depression. In a longitudinal study of a program incorporating psychotherapy, CBT, mindfulness, and a 12-step philosophy, patients seeking help for alcohol/drug dependence issues experienced significant positive changes in time perspective (Davies and Filippopoulos, 2015). This intervention facilitated a systematic review of the past and its relevance to the present. It emphasized the future possibility of positive change. Some psychologists have employed a practical form of time perspective therapy (TPT). This treatment is a version of narrative therapy, used in PTSD treatment. It has been applied with considerable success (Sword et al., 2014). The TPT therapist works to replace the negative perspective with a new narrative focused on identifying positive aspects of the patient's past, creating a favorable attitude toward the present, and constructing a vibrant image of a hopeful future. Tailored psychotherapy for the reconstruction of time perspective should be considered during interventions in addiction treatment. Besides, a study in China also indicates that the practices of meditation can improve dispositional mindfulness, resilience, and inner peace, thus significantly reducing past negative time perspectives as well as the symptoms of mental health problems, such as PTSD and depression (Ge et al., 2020).

Treatment for AD with concurrent depression requires the proper use of medication, efficacious psychological interventions, and a commitment to treat both disorders. These results could significantly support clinicians, psychiatrists, psychologists, and those who seek to combat these disorders in finding intervention strategies to address comorbidity between depression and AD.

### Limitation

The study has a few limitations. First, our study focused specifically on patients with AD. Thus, our findings should be generalized to other populations with the utmost caution, and future studies would be more comprehensive when including a control group. Second, we assessed participants' symptoms using psychometric questionnaires, which could have led to self-report bias. Although we used a specific Chinese version of ZTPI, additional efforts are needed to improve the inventory. Researchers have indicated that the future negative perspective should also be included in the scale (Carelli et al., 2011), especially when investigating participants characterized by negative future reviews. Compared with other well-recognized and wildly used versions of ZPTI, ZTPI-C did not have data about the values of healthy controls, so we were unable to compare our results with the normal range. Third, for the statistical methods, the analyzes described here could be strengthened by incorporating more sophisticated techniques, for example, structural equation modeling. Additionally, studies with a larger sample size would generate stronger prediction models. Furthermore, this study was cross-sectional. Therefore, any causal relationship among variables could not be established. As the predictive effects among ZPTI, depression, and AD are all bidirectional, we could not determine the exact causality from the association alone. It is likely that deviated time perspective, depression, and AD would interact reciprocally. Hence, long-term follow-up studies need to be conducted. Future longitudinal studies should observe changes in time perspective, depression, alcohol-related problems over time, whether patients relapse, and which factors influence these setbacks. It is also vital to study whether comorbidity with depression, negative perspective toward past, and FH relate to relapse.

## Conclusion

Despite the limitations described above, our study found a high comorbidity rate (59.9% ) of depression in alcohol-dependent patients. We also verified the predictive effects of temporal profile in AD, hence offering a more comprehensive understanding of the time perspective profile of patients with AD. The study demonstrates that time perspective should be taken into account during the treatment and nursing care of patients with AD. Identifying patients with biased time perspectives and providing them with tailored interventions including time-related alterations could help promote change in depression and alcohol-related problems. Moreover, we clarified the moderating roles of a PN time perspective and FH of AD in the association between depression and AD, as an enhancing effect. Screening patients with a family history of AD and a negative past perspective is valuable during treatment. We suggest that specific intervention programs tailored for patients with FH and negative perceptions of the past constitute a valid therapeutic path for addressing the comorbidity of depression and AD.

## Data availability statement

The raw data supporting the conclusions of this article will be made available by the authors, without undue reservation.

## Ethics statement

The studies involving human participants were reviewed and approved by the Affiliated Mental Health Center & Hangzhou Seventh People's Hospital, Zhejiang University School of Medicine, Hangzhou, China. The patients/participants provided their written informed consent to participate in this study.

## Author contributions

HW and XZ acquired the funding, composed the research proposal, and collected data. YZ and HW were responsible for data processing and composing the manuscript. All authors contributed to the article and approved the submitted version.

## Funding

HW and XZ were supported by Medicine and Health Science and Technology Plan Project of Zhejiang Province, China (No. 2020KY746). Open access funding provided by École Polytechnique Fédérale de Lausanne to YZ.

## Conflict of interest

The authors declare that the research was conducted in the absence of any commercial or financial relationships that could be construed as a potential conflict of interest.

## Publisher's note

All claims expressed in this article are solely those of the authors and do not necessarily represent those of their affiliated organizations, or those of the publisher, the editors and the reviewers. Any product that may be evaluated in this article, or claim that may be made by its manufacturer, is not guaranteed or endorsed by the publisher.
